# Development of a multiplex PCR assay for the simultaneous and rapid detection of six pathogenic bacteria in poultry

**DOI:** 10.1186/s13568-019-0908-0

**Published:** 2019-11-14

**Authors:** Zhihao Wang, Jiakun Zuo, Jiansen Gong, Jiangang Hu, Wei Jiang, Rongsheng Mi, Yan Huang, Zhaoguo Chen, Vanhnaseng Phouthapane, Kezong Qi, Chen Wang, Xiangan Han

**Affiliations:** 1 Shanghai Veterinary Research Institute, The Chinese Academy of Agricultural Sciences (CAAS), 518 Ziyue Road, Shanghai, 200241 People’s Republic of China; 2 College of Animal Science and Technology, Anhui Agricultural University, 130 Changjiangxilu, Hefei, 230036 People’s Republic of China; 3 Poultry Institute, Chinese Academy of Agricultural Sciences, Yangzhou, 225125 Jiangsu People’s Republic of China; 4 Biotechnology and Ecology Institute, Ministry of Science and Technology (MOST), 22797 Vientiane, Lao People’s Democratic Republic; 5 College of Animal Science and Technology, Henan University of Science and Technology, No.263 Kaiyuan Road, Luoyang, 471023 Henan People’s Republic of China

**Keywords:** Avian, Six pathogenic bacteria, Multiplex PCR assay, Specificity

## Abstract

*Escherichia coli, Pasteurella multocida, Proteus mirabilis, Pseudomonas aeruginosa, Salmonella* spp. and *Staphylococc*us *aureus* are six bacterial pathogens of avian. However, these pathogens may cause many similar pathological changes, resulting in clinical isolates that are difficult to quickly and simultaneously detect and identify. Here, a multiplex polymerase chain reaction (m-PCR) assay is reported to rapidly identify targets genes (*phoA, KMT1, ureR, toxA, invA*, and *nuc*) of these six pathogens in clinical samples. Six pairs of specific primers were designed. The optimal reaction conditions, specificity, and sensitivity of the m-PCR assay were investigated. The results showed that betaine remarkably improved amplification of the target genes. Specific test results showed that all six pathogens were detected by the proposed m-PCR protocol without cross-amplification with viruses or parasites. Sensitivity test results showed that the m-PCR system could amplify the six target genes from bacterial genomes or cultures with template amounts of 500 pg or 2.8–8.6 × 10^3^ colony forming units, respectively. Furthermore, the six bacterial pathogens isolated from the infected tissue samples were successfully identified. The proposed m-PCR assay is a useful tool to monitor and diagnose bacterial infection in birds with high specificity, sensitivity and throughput.

## Introduction

Several factors have been linked to the spread of pathogenic bacteria to poultry, including the expansion of the poultry industry, the increased mobility of humans and animals, water pollution, environmental climate change (Rodriguez-siek et al. 2005; Benskin et al. 2009). Furthermore, antibiotic administration is conventionally used for the control of bacterial diseases in poultry. However, the failure to diagnose the bacterial diseases of poultry, which may result in the misuse of antibiotic regimens and subsequent severe economic losses to the poultry industry and potential public health risks due to the consumption of contaminated poultry products (Van Den Bogaard et al. 2002).

A variety of methods have been established for the effective diagnosis of avian bacterial diseases, which include antigen-specific enzyme-linked immunosorbent assays (ELISAs), immunogold labeling and various other molecular biology techniques (Kotetishvili et al. 2002; Yano et al. 2007; Reischl 1996), especially polymerase chain reaction (PCR) technologies. However, the failure of multi-pathogen detection still was one of major deficiencies to these detection methods. For example, Park et al. (2011) established a triple PCR method for analysis of *Campylobacter* spp., *Escherichia coli* O157:H7 and *Salmonella* serotypes. Hu et al. (2011) established a triple PCR method for analysis of *Riemerella anatipestifer*, *Escherichia coli* (*E*. *coli*) and *Salmonella* with high sensitivity and specificity. Moreover, Belgrader et al. (1999) developed a rapid PCR assay that detected bacteria in 7 min and Han et al. (2011) established a loop-mediated isothermal amplification technique based on the *GroEL* gene for rapid detection of *Riemerella anatipestifer.*

Furthermore, although the most important diseases are viral in poultry, the bacterial diseases are also important, some studies have shown that the main bacterial pathogens of poultry (including avian pathogenic *Escherichia coli*, *Pasteurella multocida*, *Salmonella* spp. and *Staphylococcus aureus*) also caused severe economic losses and restricted the development of the poultry industry (Bisgaard 1993). In addition, although *Proteus mirabilis* and *Pseudomonas aeruginosa* are not considered among major bacterial pathogens for chickens, which still may spontaneously cause infection for chickens (Walker et al. 2002). More importantly, bacterial and viral infections often occur simultaneously, but the similarity of clinical signs of infected animals and the lack of high-throughput methods for the detection of pathogens, especially opportunistic species, such as *Proteus mirabilis* and *Pseudomonas aeruginosa*, have seriously hampered the control of epidemic diseases (Salmon and Watts 2000; Tanaka et al. 1995). Additionally, the sensitivity and specificity of colloidal gold detection technologies and ELISA techniques are relatively low, but yet these tests are costly. In contrast, multiplex PCR (m-PCR) can detect multiple pathogens with only one reaction with high sensitivity and specificity to distinguish between very closely related organisms, which greatly reduce costs. Hence, m-PCR is a promising tool for the efficient and accurate identification of pathogenic microbes.

To address these problems, a m-PCR assay for the simultaneous and rapid detection of six bacterial pathogens of poultry was developed in this study. The m-PCR assay showed high specificity, sensitivity and throughput, which should facilitate the prevention and rapid diagnosis of avian bacterial diseases.

## Materials and methods

### Bacterial strains and growth conditions

Six pathogenic bacteria were isolated from diseased birds (Table 1). The bacterial, viruses and parasites were preserved in our laboratory (Table 1). *Pasteurella multocida* was cultured in sterile Martin broth medium (Qingdao Hope Bio-Technology Co., Ltd., Qingdao, Shandong, China) overnight at 37 °C. *Streptococcus suis* was cultured in sterilized Todd-Hewitt broth (BD Medical Technology Ltd., New Jersey, USA) at 37 °C. *Listeria monocytogenes* were cultured in Brain–Heart Infusion broth (BD Medical Technology Ltd., New Jersey, USA) at 37 °C. *Escherichia coli*, *Proteus mirabilis*, *Pseudomonas aeruginosa*, *Salmonella* spp. and *Staphylococcus aureus* and the other bacteria were all cultured overnight at 37 °C in sterilized Luria–Bertani (LB) broth (Oxoid Ltd., Hampshire, UK).

**Table 1:** Pathogens used in this study

Pathogens	Species	Description	Serial number	Source
Bacterial pathogens	*Escherichia coli*	*Escherichia coli* wild-type stain from duck, serotype O_2_	CGMCC10601	CGMCC
	*Escherichia coli*	*Escherichia coli* wild-type stain from duck, serotype O_1_	APEC O1	This study
	*Escherichia coli*	*Escherichia coli* wild-type stain from duck, serotype O_78_	APEC94	This study
	*Salmonella* pullorum	*Salmonella enterica* serovar pullorum strain from poultry	ATCC10398	ATCC
	*Salmonella typhimurium*	*Salmonella enterica* serovar *typhimurium* strain from poultry	SL14028	This study
	*Salmonella enteritidis*	*Salmonella enterica* serovar *enteritidis* strain from poultry	ATCC13076	ATCC
	*Staphylococc*us *aureus*	*Staphylococc*us *aureus* wild-type from avian	ATCC29213	ATCC
	*Pseudomonas aeruginosa*	*Pseudomonas aeruginosa* wild-type strain from chicken	SHCPa120	This study
	*Proteus mirabilis*	*Proteus mirabilis* wild-type strain from goose	AHGPm101	This study
	*Pasteurella multocida*	*Pasteurella multocida* wild-type strain from chicken	Pm01	This study
	*Klebsiella pneumoniae*	*Klebsiella pneumoniae* wild-type strain form chicken	CMCC46117	This study
	*Shigella flexneri*	*Shigella flexneri* wild-type strain	CMCC51572	This study
	*Bacillus subtilis*	*Bacillus subtilis* wild-type strain	ATCC6633	This study
	*Bacillus cereus*	*Bacillus cereus* wide-type strain from chicken	CMCC63303	CMCC
	*Enterococcus faecalis*	*Enterococcus faecalis* wide-type strain from chicken	ATCC29212	CMCC
	*Listeria monocytogenes*	*Listeria monocytogenes* wide-type strain from rabbit	ATCC15313	ATCC
	*Streptococcus suis*	*Streptococcus suis* wide-type strain from pig	HA9801	CMCC
Parasite pathogens	*Cryptosporidium baileyi*	Preserved in laboratory (isolated from chicken)	AUCP-1	ATCC
	*Eimeria tenella*	Preserved in laboratory (isolated from chicken)	CAAS2111160721	ATCC
Virus pathogens	*Newcastle disease virus*	Newcastle disease virus strain Lasota (isolated form avian)	JF950510	This study
	*Infectious bursal disease*	Preserved in laboratory (isolated from chicken)	NF8	This study
	*Avian influenza H9N2*	Preserved in laboratory (isolated from duck)	2011 (H9N2)	This study

*CGMCC* China General Microbiological Culture Collection Center, *ATCC* American Type Culture Collection, *CMCC* China Medical Microbial Culture Collection Management Center

All bacteria were cultured until the mid-log phase, and then the bacterial genomes were extracted according to the previous methods (Velegraki et al. 1999) with some modifications. The genomes of the parasites and viruses were preserved in our laboratory.

The enzymes Ex Taq polymerase (Mg^2+^ free) (Lot#KA7201HA), loading buffer (Lot#KA701A), and DNA Maker (Lot#A2301A) were purchased from TaKaRa Biotechnology Co., Ltd. (Dalian, China). A genome extraction kit was purchased from Tiangen Biotech Co., Ltd. (Beijing, China). Betaine was purchased from Sigma-Aldrich Corporation (St. Louis, MO, USA).

### Numbers of colony forming units (CFU) of the six pathogens

After culturing of the six pathogenic bacteria overnight on agar plates, the cells were collected and washed twice with phosphate-buffered saline. The optical density at 600 nm (OD_600_) of the bacterial suspensions was adjusted to 1, then 10^4^-, 10^5^-, 10^6^-, and 10^7^-fold dilutions were prepared. Aliquots (2 μL) of the bacteria solution were placed in agar plates, which were cultured overnight at 37 °C. After 12 h, the CFUs of six pathogens (OD_600_ = 1.0) were counted, respectively.

### Design of primers and amplification of target genes

In this study, m-PCR assay primers were designed with Primer premier 5.0 software (Premier Biosoft International, Palo Alto, CA, USA) according to the conserved regions of the following target genes: *Escherichia coli phoA* gene (NC_000913.3), *Pasteurella multocida KMT1* gene (NZ_CP008918.1), *Proteus mirabilis ureR* gene (NC_010554.1), *Pseudomonas aeruginosa toxA* gene (CP017306.1), *Salmonella* spp. *invA* gene (AE014613.1), and *Staphylococcus aureus* gene (AP017922.1).

The sequences of the *phoA*, *KMT1*, *ureR*, *toxA*, *invA*, and *nuc* genes were obtained from the GenBank database (http://www.ncbi.nlm.nih.gov/Genbank/). All oligonucleotide primers used in this study were synthesized by Shanghai Sunny Biotechnology Co., Ltd. (Shanghai, China). The sequences of the PCR primers are shown in Table 2.

**Table 2:** Primers used in this study

Species	Primers	Sequence	Products (bp)
*Escherichia coli*	phoA P1	GCACTCTTACCGTTACTGTTTACCCC	1001
	phoA P2	TTGCAGGAAAAAGCCTTTCTCATTTT	
*Pasteurella multocida*	KMT1 P1	TTAACAGAGAGGTGAAAAATACCCCTA	755
	KMT1 P2	CTTTACGCTGATTAATATTGTGCTGA	
*Proteus mirabilis*	ureR P1	CTGGTGGCTCATTCATCT	509
	ureR P2	ACAGTTAGGCGGTGGTAT	
*Pseudomonas aeruginosa*	toxA P1	TTCGTCAGGGCGCACGAGAGCA	363
	toxA P2	TCTCCAGCGGCAGGTGGCAAG	
*Salmonella* spp.	invA P1	AACCAGCAAAGGCGAGCAG	256
	invA P2	AATACGATGCTGTTATCGTCCAG	
*Staphylococcus aureus*	nuc P1	CCTGAAACAAAGCATCCTAAAAA	155
	nuc P2	TAAATATACGCTAAGCCACGTCCAT	

In order to evaluate and verify the specificity of the primers, PCR analysis was performed using the genomes of the six pathogens as DNA templates.

### Optimization of m-PCR primers

Optimization of the primer combinations was based on the orthogonal experimental method. In the 15 double combinations, the optimal combination was selected as the initial double PCR and the remaining four primer pairs (the initial concentration of each primer was 0.4 µM) were added to the double combination to form a triple PCR. An optimal triple PCR was then selected and the remaining three primer pairs were added to form a quadruple PCR, until completion of the m-PCR.

After the addition of a new primer pair to an optimal PCR, if the combination was not optimal, the primers were redesigned, and then the concentration of each primer was adjusted from initial concentration of 0.4 µM to achieve the best results.

### Optimization of m-PCR conditions

The PCR reaction is affected by many factors. Therefore, the parameters of the m-PCR assay were optimized by varying concentration of deoxyribonucleotide triphosphate (dNTPs; 0.1–0.4 mM), Mg^2+^ (0.2–0.5 mM), Taq DNA polymerase (1.0, 1.5, 2.0, and 2.5 U), and betaine (0.05–0.4 mM) in a 25-µL reaction volume.

A mixture of the genomic DNA, which contained same amount of genomic DNA of the six types of bacteria, was used as a template to amplify the corresponding target genes. The total volume of each reaction system (recommended system) was 25 μL, which included 1 μL of template DNA (about 150 ng of genomic DNA).

PCR cycles were as follows: pre-denaturation at 94 °C for 4 min, denaturation at 94 °C for 40 s, annealing at 58 °C for 30 s, extension at 72 °C for 1 min, for 25–35 cycles, extension at 72 °C for 10 min, and preservation at 16 °C. After the reaction, 5 μL of the reaction solution was mixed with 1 μL of loading buffer (6×; TaKaRa Biotechnology) for 1.5% agarose gel electrophoresis.

### Specificity of the m-PCR assay

In order to confirm the specificity of the m-PCR established in this experiment, the genomes of seven species of bacteria (including *Klebsiella pneumoniae*, *Shigella* spp., *Bacillus subtilis*, *Bacillus cereus*, *Enterococcus faecalis*, *Listeria monocytogenes* and *Streptococcus suis*), two avian parasites (*Cryptosporidium baileyi and Eimeria tenella*) and three viruses (NDV, IBDV and AIV) were selected as the DNA template for m-PCR under optimized conditions (Table 3). The m-PCR test was performed with the genomes of six bacteria as the template DNA, which served as a positive control.

**Table 3:** Composition of m-PCR system

Component	Volume (25 µL in all)
10× buffer	2.5
Ex Taq (8000 U/mL)	0.125
dNTP (2.5 mM each)	2.5
Mg^2+^ (25 mM)	2.0
phoA-P1/P2 (20 µM)	0.5
KMT1-P1/P2 (20 µM)	0.25
ureR-P1/P2 (20 µM)	0.5
toxA-P1/P2 (20 µM)	0.25
invA-P1/P2 (20 µM)	0.5
nuc-P1/P2 (20 µM)	1.0
Betaine (5 M)	2.0
Template DNA (120 ng, each)	1.0
DDH_2_O	11.875

Furthermore, different serotypes of bacterial species were selected to verify the specificity of the multiplex PCR detection system: including O_1_, O_2_ and O_78_ serotype of avian pathogenic *Escherichia coli*, *Salmonella typhimurium*, *Salmonella enteritidis* and *Salmonella pullorum* of *Salmonella* spp.

### Sensitivity of the m-PCR assay

The sensitivity of the m-PCR assay was evaluated using a tenfold serial dilution method. Briefly, the six strains were cultured to OD_600_ = 1 and then diluted to 0.1, 0.01, and 0.001, and 2 µL of the above diluents were used as PCR templates.

The six strains were cultured until the mid-logarithmic phase. After extraction, the genomes were diluted to concentrations of approximately 100 ng/μL, 75 ng/μL, 50 ng/μL, 25 ng/μL, 12.5 ng/μL, 10 ng/μL, 5 ng/μL, 1 ng/μL, 500 pg/μL, and 250 pg/μL, after which 1 µL of these diluents was tested as the m-PCR template DNA for verification.

### M-PCR for the detection of six pathogenic bacteria from experimentally or naturally infected tissue samples

The ability of the m-PCR assay to detect six pathogens in liver, spleen, and blood samples from experimentally infected chicks was evaluated. The 7-day-old San Huang chicks were obtained from Songjiang Chicken Farm (Shanghai, China) and were housed in cages under a controlled temperature of 28–30 °C and a 12 h light/dark cycle with free access to food and water during the study period. Briefly, 7-day-old San Huang chicks were injected with 5 × 10^5^ CFU of *Escherichia coli*, 5 × 10^3^ CFU of *Pasteurella multocida*, 2 × 10^8^ CFU of *Proteus mirabilis*, 1 × 10^8^ CFU of *Pseudomonas aeruginosa*, 5 × 10^4^ CFU of *Salmonella* spp. and 2 × 10^7^ CFU of *Staphylococcus aureus* in the leg muscle, respectively. Then, the liver, spleen, and blood samples were aseptically collected 24 h after injection in accordance with the guidelines of the Animal Management and Use Committee of the Shanghai Veterinary Research Institute (Chinese Academy of Agricultural Sciences). The liver, spleen, and blood samples were homogenized in phosphate-buffered saline, then cultured for 4 h in LB broth, and boiled for 5 min to extract the genomic DNA for m-PCR detection. Genomic DNA was extracted using a DNA extraction mini kit (Tiangen Biotech Co., Ltd., BeiJing, China) according to the manufacturer’s instructions. Furthermore, the 6 bacterial genome mixtures (including *Escherichia coli*, *Pasteurella multocida*, *Proteus mirabilis*, *Pseudomonas aeruginosa*, *Salmonella* spp. and *Staphylococcus aureus*) were prepared in advance as DNA template, which was used as a positive control for the m-PCR assay.

Moreover, for evaluation of the potential application of this PCR in clinical investigation, some tissue samples from diseased chicks from different poultry farms were processed during 2018–2019. The tissue samples were tested as described above.

## Results

### Amplification of target genes

**Fig. 1:**
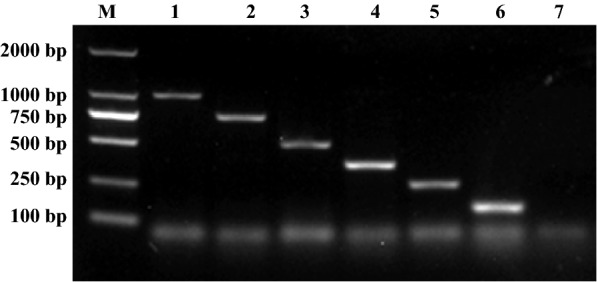
Amplification of target gene of the multiplex PCR. Lane M: 2000 bp DNA marker; Lanes 1–6: the template of m-PCR respectively were *Escherichia coli, Pasteurella multocida, Proteus mirabilis, Pseudomonas aeruginosa, Salmonella* spp. and *Staphylococcus aureus*; Lane 7: negative control

The designed primers successfully amplified 1001 bp of the *Escherichia coli PhoA* gene (Fig. 1, lane 1), 755 bp of the *Pasteurella multocida KMT1* gene (Fig. 1, lane 2), 509 bp of the *Proteus mirabilis ureR* gene (Fig. 1, lane 3), 363 bp of the *Pseudomonas aeruginosa toxA* gene (Fig. 1, lane 4), 256 bp of the *Salmonella* spp. *invA* gene (Fig. 1, lane 5), and 155 bp of the *Staphylococcus aureus nuc* gene (Fig. 1, lane 6). Different sizes of the PCR products of each target gene were produced for size discrimination by agarose gel electrophoresis.

### Number of CFUs of the six pathogens

The plate counting results showed that amounts of *Escherichia coli, Pasteurella multocida*, *Proteus mirabilis*, *Pseudomonas aeruginosa*, *Salmonella* spp. and *Staphylococcus aureus* were 5.00 × 10^8^, 3.08 × 10^8^, 1.41 × 10^9^, 4.28 × 10^9^, 1.88 × 10^9^, and 2.79 × 10^9^ CFU at OD_600_ of 1.0, respectively. The results of three independent experiments were similar.

### Optimization of the m-PCR primers

**Fig. 2:**
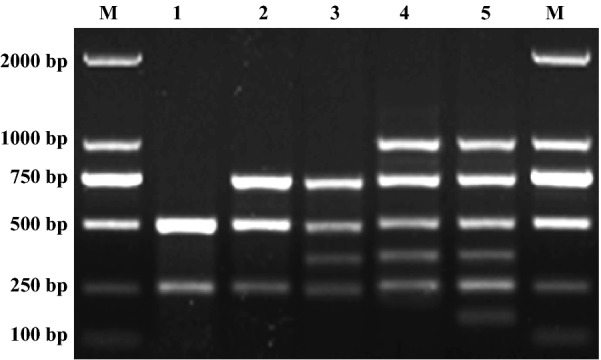
Optimization of the primers of m-PCR. Lane M: 2000 bp DNA marker; Lane 1: double PCR formed by *Proteus mirabilis* and *Salmonella* spp.; Lane 2: triple PCR formed by *Proteus mirabi*lis, *Salmonella* spp. and *Pasteurella multocida*; Lane 3: quadruple PCR formed by *Proteus mirabi*lis, *Salmonella* spp., *Pasteurella multocida* and *Pseudomonas aeruginosa*; Lane 4: quintuple PCR formed by *Proteus mirabi*lis, *Salmonella* spp., *Pasteurella multocida*, *Pseudomonas aeruginosa* and *Escherichia coli*; Lane 5: sextuple PCR formed by *Proteus mirabi*lis, *Salmonella* spp., *Pasteurella multocida*, *Pseudomonas aeruginosa*, *Escherichia coli* and *Staphylococcus aureus*

As shown in Fig. 2, of the 15 double combinations, the optimal combination of *Proteus mirabilis* and *Salmonella* spp. (Fig. 2, lane 1) was selected for the initial double PCR assay. Subsequently, a third primer pair was added to form a triple PCR. For the triple m-PCR assay, the combination of *Proteus mirabilis*, *Salmonella* spp. and *Pasteurella multocida* (Fig. 2, lane 2) was optimal. According to the orthogonal experiments, quadruple, quintuple, and sextuple m-PCR assays were successively established (Fig. 2, lanes 3, 4, and 5, respectively).

### Optimization of the m-PCR conditions

The results showed that the optimal annealing temperature of the m-PCR reaction was 54 to 58 °C, while the optimal dNTP and Mg^2+^ concentrations were 0.1 mM and 2.5 mM, respectively (data not shown). The optimum betaine concentration was 0.4 M (data not shown).

In addition, the concentrations of each pair of primers were optimized based on the orthogonal experimental method, the results showed that the optimal concentrations of each pair of oligonucleotide primers were 0.2 µM (*Pseudomonas aeruginosa and Pasteurella multocida*), 0.4 µM (*Proteus mirabilis*, *Salmonella* spp. and *Escherichia coli*) and 0.8 µM (*Staphylococcus aureus*), respectively (data not shown).

**Fig. 3:**
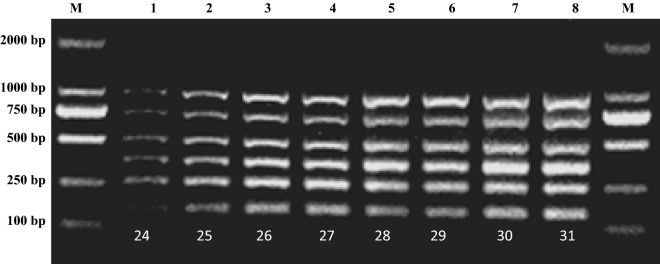
Determination of time of the multiplex PCR. Lane M: 2000 bp DNA marker; Lane 1: 24 running cycles; Lane 2: 25 running cycles; Lane 3: 26 running cycles; Lane 4: 27 running cycles; Lane 5: 28 running cycles; Lane 6: 29 running cycles; Lane 7: 30 running cycles; Lane 8: 31 running cycles

Furthermore, the number of cycles largely determines the required total duration of the m-PCR assay. The optimal number of m-PCR cycles was 25, which is considerably shorter the normally required 30–35 cycles (Fig. 3).

### Specificity of the m-PCR assay

**Fig. 4:**
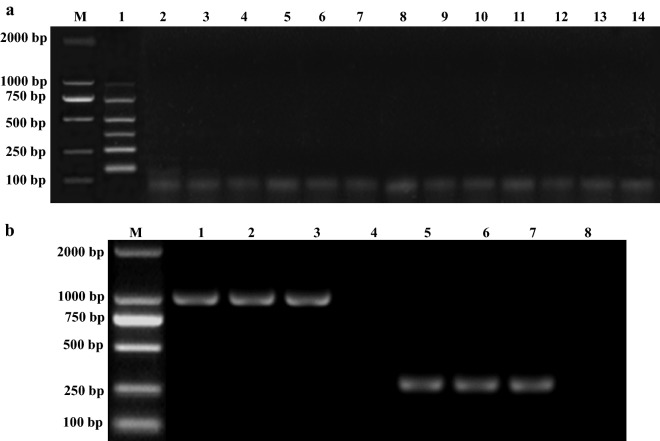
Determination of specificity of the multiplex PCR. Lane M: 2000 bp DNA marker; **a** Lane 1: the template of m-PCR contain 6 bacterial genomes as positive control. Lane 2: the template of m-PCR was *Klebsiella pneumoniae*; Lane 3: the template of m-PCR was *Shigella* spp.; Lane 4: the template of m-PCR was *Bacillus subtilis*; Lane 5: the template of m-PCR was *Bacillus cereus*; Lane 6: the template of m-PCR was *Enterococcus faecalis*; Lane 7: the template of m-PCR was *Listeria monocytogenes*; Lane 8: the template of m-PCR was *Streptococcus suis*; Lane 9: the template of m-PCR was *Cryptosporidium baileyi*; Lane 10: the template of m-PCR was *Eimeria tenella*; Lane 11: the template of m-PCR was Newcastle disease virus (NDV); Lane 12: the template of m-PCR was Avian Influenza virus H9N2; Lane 13: the template of m-PCR was infectious bursal disease virus (IBDV); Lane 14: Negative control. **b** Lanes 1–3: the template of m-PCR was O_1_, O_2_ and O_78_ serotype of avian pathogenic *Escherichia coli*, respectively; Lanes 5–7: the template of m-PCR was *Salmonella typhimurium*, *Salmonella enteritidis*, *Salmonella pullorum,* respectively; Lanes 4, 8: negative control

The results showed that oligonucleotide primers specific for the *phoA*, *KMT1, ureR, toxA, invA* and *nuc* genes produced amplification products with sizes of 1001, 755, 509, 363, 256, and 155 bp, respectively. The addition of DNA from *Klebsiella pneumoniae* (Fig. 4a, lane 2), *Shigella flexneri* (Fig. 4a, lane 3), *Bacillus subtilis* (Fig. 4a, lane 4), *Bacillus cereus* (Fig. 4a, lane 5), *Enterococcus faecalis* (Fig. 4a, lane 6), *Listeria monocytogenes* (Fig. 4a, lane 7), *Streptococcus suis* (Fig. 4a, lane 8), *Cryptosporidium baileyi* (Fig. 4a, lane 9), *Eimeria tenella* (Fig. 4a, lane 10), Newcastle disease virus (NDV) (Fig. 4a, lane 11), Infectious bursal disease virus (IBDV) (Fig. 4a, lane 12) and Avian Influenza virus H9N2 (Fig. 4a, lane 13) as PCR templates did not amplify the corresponding sizes of PCR product bands.

Furthermore, the results also indicated that different serotypes of avian pathogenic *Escherichia coli* (Fig. 4b, lanes 1–3) or *Salmonella* spp. (Fig. 4b, lanes 5–7) could be detected by m-PCR assay.

### Sensitivity of the m-PCR assay

**Fig. 5:**
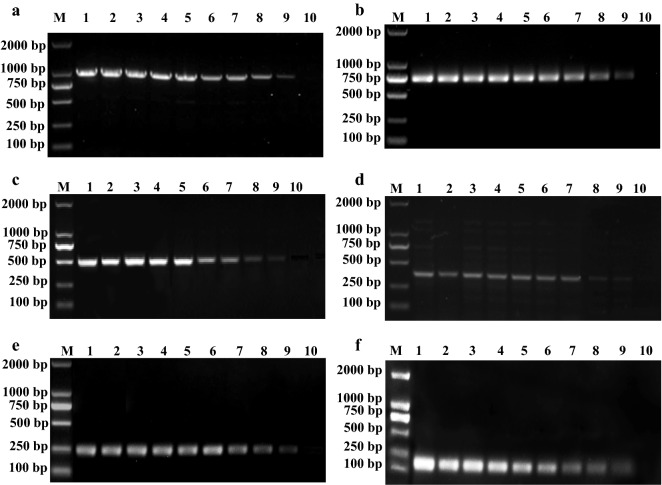
Determination of the sensitivity of the multiplex PCR for bacterial genomic DNA detection. Lane M: 2000 bp DNA marker; **a** Lanes 1–10: the concentration of *Escherichia coli* DNA were 100 ng, 75 ng, 50 ng, 25 ng, 12.5 ng, 7.5 ng, 2.5 ng, 1 ng, 500 pg and 250 pg, respectively. b Lanes 1–10: the concentration of *Pasteurella multocida* DNA were 100 ng, 75 ng, 50 ng, 25 ng, 12.5 ng, 7.5 ng, 2.5 ng, 1 ng, 500 pg and 250 pg, respectively. **c** Lanes 1-8: the concentration of *Proteus mirabilis* DNA were 100 ng, 75 ng, 50 ng, 25 ng, 12.5 ng, 7.5 ng, 2.5 ng, 1 ng, 500 pg and 250 pg, respectively. **d** Lanes 1–10: the concentration of *Pseudomonas aeruginosa* DNA were 100 ng, 75 ng, 50 ng, 25 ng, 12.5 ng, 7.5 ng, 2.5 ng, 1 ng, 500 pg and 250 pg, respectively. **e** Lanes 1–10: the concentration of *Salmonella* spp. DNA were 100 ng, 75 ng, 50 ng, 25 ng, 12.5 ng, 7.5 ng, 2.5 ng, 1 ng, 500 pg and 250 pg, respectively. **f** Lanes 1–10: the concentration of *Staphylococcus aureus* DNA concentration were 100 ng, 75 ng, 50 ng, 25 ng, 12.5 ng, 7.5 ng, 2.5 ng, 1 ng, 500 pg and 250 pg, respectively

**Fig. 6:**
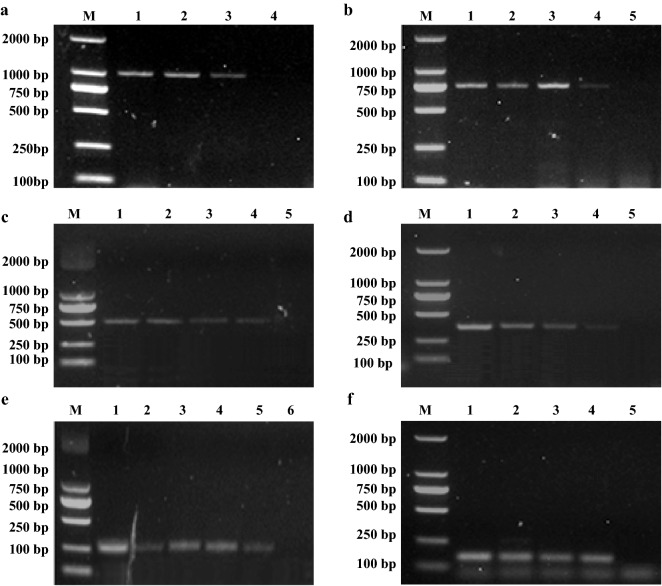
Determination of the sensitivity of the m-PCR for bacterial CFU detection. Lane M: 2000 bp DNA Marker. **a** Lanes 1–4: the template of *Escherichia coli* respectively were 5 × 10^5^, 5 × 10^4^, 5 × 10^3^ and 5 × 10^2^ CFU, respectively. **b** Lanes 1–5: the template of *Pasteurella multocid*a respectively were 6 × 10^6^, 6 × 10^5^, 6 × 10^4^, 6 × 10^3^ and 6 × 10^2^ CFU, respectively. **c** Lanes 1–5: the template of *Proteus mirabilis* respectively were 2.8 × 10^6^, 2.8 × 10^5^, 2.8 × 10^4^, 2.8 × 10^3^ and 2.8 × 10^2^ CFU, respectively. **d** Lanes 1–5: the template of *Pseudomonas aeruginosa* respectively were 8.6 × 10^6^, 8.6 × 10^5^, 8.6 × 10^4^, 8.6 × 10^3^, 8.6 × 10^2^ CFU, respectively. **e** Lanes 1–6: the template of *Salmonella* spp. respectively were 3.2 × 10^7^, 3.2 × 10^6^, 3.2 × 10^5^, 3.2 × 10^4^, 3.2 × 10^3^ and 3.2 × 10^2^ CFU, respectively. **f** Lanes 1–5: the template of *Staphylococcus aureus* respectively were 5.6 × 10^6^, 5.6 × 10^5^, 5.6 × 10^4^, 5.6 × 10^3^ and 5.6 × 10^2^ CFU, respectively

The detection limits of the genomic DNA concentrations for *Escherichia coli* (Fig. 5a), *Pasteurella multocida* (Fig. 5b)*, Proteus mirabilis* (Fig. 5c), *Pseudomonas aeruginosa* (Fig. 5d), *Salmonella* spp. (Fig. 5e) and *Staphylococcus aureus* was all about 500 pg (Fig. 5f), respectively. The detection limits of CFUs of *Escherichia coli* was 5 × 10^3^ (Fig. 6a), *Pasteurella multocida* was 6 × 10^3^ (Fig. 6b), *Proteus mirabilis* was 2.8 × 10^3^ (Fig. 6c), *Pseudomonas aeruginosa* was 8.6 × 10^3^ (Fig. 6d), *Salmonella* spp. was 3.2 × 10^3^ (Fig. 6e) and *Staphylococcus aureus* was 5.6 × 10^3^ (Fig. 6f), respectively. All experiments were conducted in triplicate.

### M-PCR analysis of experimentally or naturally infected tissue samples

As shown by the results of experimentally infected tissue samples in Table 4, all pathogens can be detected in the liver samples, while *Escherichia coli*, *Pasteurella multocida* and *Salmonella* spp. were detected in the blood samples, and all, except for *Proteus mirabilis*, were detected in the kidney samples.

**Table 4:** M-PCR detection filtrates from tissues and organs after enrichment for 4 h

Species	Liver	Kindey	Blood	Detected
*Escherichia coli*	+	+	+	+
*Pasteurella multocida*	+	+	+	+
*Proteus mirabilis*	+	−	−	+
*Pseudomonas aeruginosa*	+	−	+	+
*Salmonella* spp.	+	+	+	+
*Staphylococcus aureus*	+	−	+	+

+, can be detected

−, can’t be detected

Besides, for evaluation of the potential application of this PCR in clinical investigation, some samples of chicks from natural outbreaks were processed during 2018–2019. These results of detection of natural infections samples showed 82 strains of *Escherichia coli*, 6 strains of *Pasteurella multocida*, 40 strains of *Proteus mirabilis*, 2 strains of *Pseudomonas aeruginosa*, 30 strains of *Salmonella* spp. and 5 strains of *Staphylococcus aureus* were identified and isolated by the assay, respectively (data not show).

The results indicated that the assay can provide specific detection of six pathogenic bacteria in experimentally or naturally infected tissue samples.

## Discussion

Bacterial infection remains an important issue in the poultry industry (Cox and Pavic 2010) because of the huge economic losses due to infectivity, high mortality, and widespread drug resistance. Furthermore, the clinical signs several different bacterial pathogens are very similar and is very difficult to identified the agent without laboratorial analyses. For example, although *Proteus mirabilis* and *Pseudomonas aeruginosa* were conditional pathogens, which still could cause respiratory diseases (Walker et al. 2002), while avian pathogenic *Escherichia coli* and *Pasteurella multocida* cause high mortality in chicks (Dho-moulin and Fairbrother 1999). Hence, development an m-PCR assay with high specificity, sensitivity and throughput would be very useful for monitoring and diagnose bacterial infections in birds. However, at present, there is no molecular method for the simultaneous detection of the six major pathogens of chickens (*Escherichia coli*, *Pasteurella multocida*, *Proteus mirabilis*, *Pseudomonas aeruginosa*, *Salmonella* spp. and *Staphylococcus aureus*).

Primer specificity is a critical determinant of the success of an m-PCR assay. In this study, an m-PCR assay was developed to target specific genes of six pathogens (*invA, phoA, KMT1, toxA, ureR*, and *nuc*) based on the following previous studies. Rahn et al. (1992) reported the use of the *invA* gene for specific detection of *Salmonella* spp. Thong et al. (2011) established an m-PCR assay for detection of the *phoA* gene of *Escherichia coli.* Townsend et al. (1998) and Blackall and Miflin (2000) developed PCR assays for identification of the *KMT1* gene of *Pasteurella multocida*. Song et al. (2000) designed specific primers for the rapid identification of the *Pseudomonas aeruginosa toxA* gene. The *Proteus mirabilis ureR* gene was identified as a transcriptional regulator of the urease enzyme (Nicholson et al. 1993) and has been used as target gene for the detection of *Proteus mirabilis* by PCR (Huang et al. 1999). Brakstad and Maeland (1995) established a method for the direct identification of the *Staphylococcus aureus nuc* gene.

Further, to test cross reaction by agents that could be found as secondary infection with avian parasites (cryptosporidium and coccidia) and viruses (NDV, IBDV and AIV) showed that the proposed m-PCR assay had very high specificity. Moreover, the specificity also was tested by the different major serotype of the avian pathogenic *Escherichia coli* and *Salmonella* spp. which were the most important bacterial pathogens of poultry according to the clinical isolation samples. In addition, in poultry infections, the major serotype of the other bacteria such as *Pasteurella multocida, Proteus mirabilis, Pseudomonas aeruginosa and Staphylococcus aureus* is single.

In most cases, the sensitivity of an m-PCR assay will be reduced with increased numbers of target genes in the system. However, the detection limit of the proposed m-PCR assay was 2.8–8.6 × 10^3^ CFU of each bacterial species, which is in agreement with the results of previous studies. For example, the sensitivity of *Escherichia coli* detection with the proposed m-PCR assay was 10^3^ CFU/mL, which was superior to that reported by Kong et al. (2002) of 10^4^ CFU/mL. The detection limits of *Proteus mirabilis* and *Pasteurella multocida* were 8.6 × 10^3^ and 2.8 × 10^3^ CFU/mL, which were the same orders of magnitude as those reported by Huang et al. (1999), Takeuchi et al. (1996). On the contrary, previously reported PCR assays for the detection of these six pathogens were single, triple, or quadruple methods. In comparison, the PCR detection sensitivities established in this study were close to or even exceeded those of the cited PCR assays. Moreover, as compared with traditional detection methods, the six pathogens tested in this study can be detected at one time with high sensitivity, thereby greatly reducing the detection time, while improving the efficiency.

For optimization of the m-PCR assay, the concentrations of primers, Taq DNA polymerase, and dNTPs, as well as the addition of a PCR additive, were optimized in this study. In pre-experiments, the concentrations of the first primer pairs for *Proteus mirabilis* and *Salmonella* spp. were set at 0.4 µM. For the following orthogonal experiments, a third pair of primers was added and the concentration was adjusted from 0.1 to 0.8 µM until the specificity was judged as appropriate.

dNTPs are raw materials for the synthesis of target fragments. Hence, to synthesize larger target fragments, more dNTPs are consumed. In this study, the target fragments were all within 1000 bp, but six were synthesized. Therefore, under consideration of cost, we recommend a dNTP concentration of 0.25 mM to amplify the corresponding target fragments.

Betaine is widely used as an enhancer to optimize various PCR assays. For example, Marshall et al. (2015) used betaine to enhance the formation of long PCR products and Henke et al. (1997) reported that betaine improved the amplification of genes by reducing the formation of secondary structures caused by GC-rich regions. As compared to dithiothreitol and dimethyl sulfoxide, betaine had the best PCR enhancing properties at a concentration of 0.8 M for all primer pairs and was more effective since the PCR output was enhanced for all of the target fragments (Hengen 1997; Kang et al. 2005; Lajin et al. 2013). The major limitation of detection is a low quantity of the template. In this study, the addition of 0.4 M betaine improved the sensitivity of the PCR assay so that the detection limit of the sextuple PCR assay was similar to that of a single assay.

In addition, to determine whether the m-PCR assay was appropriate for the detection of pathogens in clinical and laboratory samples, 7-day-old chicks were inoculated with the six tested pathogens. Then the pathogens were enriched from the tissues and organs of chicks for detection by m-PCR. The results showed that all six pathogens were detected in the liver samples with the proposed m-PCR assay. Besides, more than 150 bacterial strains were identified and isolated from diseased chicken by the assay. The results indicated that the assay also can be used in clinical investigation.

In conclusion, a rapid diagnostic m-PCR assay was established for the detection of six pathogenic bacteria in a short time. Moreover, this method can effectively and rapidly detect most pathogenic bacterial infections in poultry with good specificity, accuracy, and sensitivity.

## Data Availability

The data on which the conclusions are made are all presented in this paper.

## Abbreviations

m-PCR: multiplex polymerase chain reaction
ELISAs: enzyme-linked immunosorbent assays
LB: Luria–Bertani
CFU: colony forming units
